# Sleep disorder, Mediterranean diet, and all-cause and cause-specific mortality: a prospective cohort study

**DOI:** 10.1186/s12889-023-15870-x

**Published:** 2023-05-18

**Authors:** Yongle Wang, Hongxuan Fan, Zhaoyu Ren, Xuchang Liu, Xiaoyuan Niu

**Affiliations:** 1 Department of Neurology, First Hospital of Shanxi Medical University, No. 85, Jiefangnan Street, Yingze District, Taiyuan City, 030001 Shanxi Province China; 2 Clinical college, Shanxi Medical University, No. 58, Xinjiannan Street, Yingze District, Taiyuan City, Shanxi Province China; 3 Department of Cardiology, Second Hospital of Shanxi Medical University, Taiyuan City, Shanxi Province China

**Keywords:** Mediterranean diet, Sleep disorder, All-cause mortality, NHANES, Cardiovascular mortality

## Abstract

**Background:**

There is a bidirectional effect between sleep disorders and Mediterranean diet (MED), but the joint effect of MED and sleep disorders on mortality is unclear. The aim of this study was to investigate whether there is a synergistic effect of adherence to MED and sleep disorders on all-cause and cause-specific mortality.

**Methods:**

The study included 23,212 individuals in the National Health and Nutrition Examination Survey (NHANES) from 2005 to 2014. A 9-point evaluation score, alternative Mediterranean diet (aMED) index was used to assess adherence to MED. Sleep disorder and hours of sleep were assessed by structured questionnaires. Cox regression models were used to assess the relationship between sleep disorders, aMED and all-cause mortality, cause-specific mortality (cardiovascular-related death, cancer-related death). The interaction effect of sleep disorders with aMED on mortality was further assessed.

**Results:**

Results showed that participants with lower aMED and presence of sleep disorders had significantly higher risk of all-cause mortality and cardiovascular-related mortality (HR, 2.16, 95% CI, 1.49–3.13, P < 0.0001; HR, 2.68, 95% CI, 1.58–4.54, P = 0.0003). A significant interaction effect was found between aMED and sleep disorders on cardiovascular mortality (p for interaction = 0.033). No significant interaction existed between aMED and sleep disorders on all-cause mortality (p for interaction = 0.184) and cancer-related mortality (p for interaction = 0.955).

**Conclusions:**

Poorer adherence to MED and sleep disorders synergistically increased long-term all-cause mortality and cardiovascular mortality in NHANES population.

**Supplementary Information:**

The online version contains supplementary material available at 10.1186/s12889-023-15870-x.

## Introduction

Numerous studies have confirmed that poor dietary habits may increase the risk of chronic diseases and mortality risk in the long term [1]. The potential mechanisms may include elevated inflammation and oxidative stress levels, deficiencies in essential nutrients, and irregular meal times [2]. On the other hand, a high-quality dietary pattern characterized by low inflammation, low sugar, and high dietary fiber can lower the incidence of cardiovascular diseases and cancer, thereby reducing long-term mortality rates [3, 4]. The Mediterranean diet (MED) is characterized as rich in fruits, vegetables, whole grains, nuts, legumes and fish, as well as healthy unsaturated fatty acids, vitamins and trace elements [5, 6] MED has been widely recognized as beneficial to the cardiovascular system, reducing the risk of long-term cardiovascular disease (CVD)[7]. The MED also has anti-cancer effects. Several studies have found that the MED reduces the risk of certain types of cancer, including breast, colorectal, stomach, and prostate cancers [8, 9] This may be due to that MED is rich in antioxidants and other beneficial components that help protect cells from free radical damage, which in turn reduces the formation and growth of cancer cells [10, 11] The MED has been shown to reduce the risk of cardiovascular mortality, cancer mortality, and all-cause mortality [12–16]. Relevant evidence suggests that the beneficial effects of MED may be associated with its ability to lower inflammation levels, oxidative stress levels, and improve metabolic disorders.

On the other hand, there are growing evidences on the relationship between sleep disorders and all-cause mortality, but the conclusions are controversial. The general opinion is that both shorter and longer sleep duration increase the risk of mortality and that sleep duration shows a U-shaped relationship with long-term mortality [17] In addition, insomnia, difficulty in falling/maintaining sleep and early awakening may increase the risk of CVD and are associated with all-cause mortality and CVD mortality [18–21] However, previous studies investigated the issue by focusing on ethnic-specific or disease-specific populations, and cohort studies on the long-term prognosis of sleep disorder to all-age and all-race populations are lacking. In addition, there is a significant correlation between diet and sleep, affecting each other [22] Inadequate and irregular sleep pattern may lead to overfeeding and poorer dietary structure [22] Also, varied dietary habits, and food components can have an impact on sleep disorders/parameters [23] Certain fruits, vegetables and fish rich in melatonin or its precursor components (e.g. tryptophan, serotonin) can improve sleep quality in the general population [24] The MED has been shown to significantly improve sleep disorders [25] On the contrary, foods rich in added sugar, caffeine, and saturated fatty acids can affect the quality and duration of sleep [26–28] However, these studies are limited to cross-sectional studies, and cohort studies of MED and sleep disorders on long-term prognostic outcomes, such as risk of cardiovascular events and mortality, are lacking.

In summary, the effect of MED and sleep on death in the whole population needs to be further clarified. Furthermore, given that diet and sleep can affect each other, it is unclear if lower adherence level to MED can interact with sleep disorders for long-term mortality. Therefore, the article investigated whether there is a synergistic effect of adherence to MED and sleep disorders on all-cause and cause-specific mortality in U.S. population.

## Methods

### Study population

**Fig. 1:**
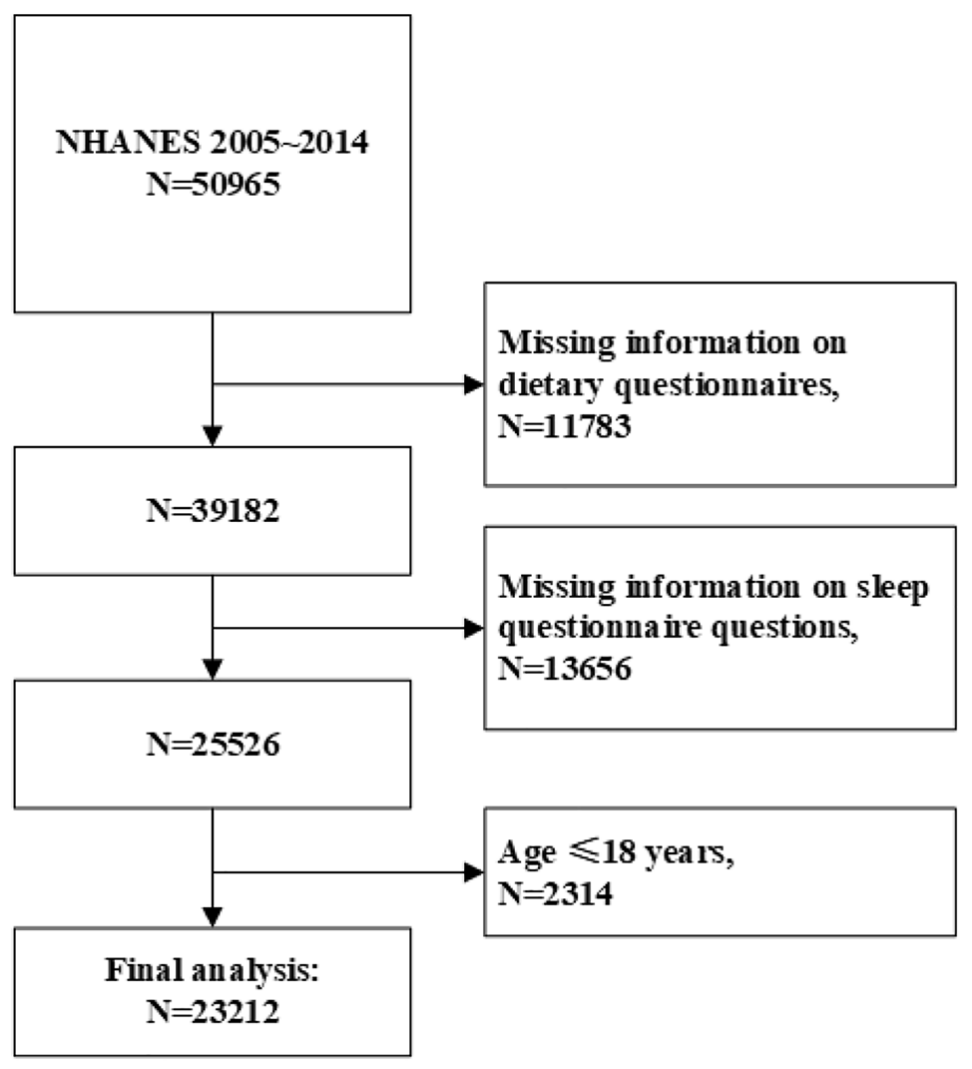
Flowchart of the study inclusion and exclusion of participant. Participants were excluded due to missing information on dietary and sleep questionnaires, aged less than 18 years. A total of 23,212 patients were included finally

NHANES is a national, cross-sectional survey that assesses the health and nutritional status of the U.S. population. Based on a complex sampling design, the population included in this survey is representative of the U.S. population of all ages. The survey includes interview questionnaires, standardized physical examinations, and laboratory tests [29] The survey was approved by the National Center for Health Statistics (NCHS) Institutional Review Board (https://www.cdc.gov/nchs/nhanes/irba98.htm) and informed consent was obtained from all participants before enrollment (https://www.cdc.gov/nchs/nhanes/genetics/genetic_participants.htm#print). The study was conducted in accordance with the Declaration of Helsinki. Data obtained from five cycles (every two years) from 2005 to 2014 were included in this study. Follow-up data on death status, death date and causes of death were linked to the National Death Index (NDI) (https://www.cdc.gov/nchs/ndi/about.htm). The NDI, maintained by NCHS, is published annually and provides epidemiologists with complete population-wide mortality data for the U.S.[30] The inclusion and exclusion process of the study population is shown in Fig. 1. Patients aged ≤ 18 years, who did not complete the dietary recall questionnaire, the sleep questionnaire, or who were lost to follow-up were excluded. Ultimately, there were 23,212 individuals included in the final analysis. The average age of the participants included was 46.6 years, with 52.5% being female. The majority of the participants were of non-Hispanic white race, accounting for 67.6% of the total sample.

### Data collection

#### Assessment of aMED and sleep disorder

Relevant dietary information was collected from the 24-h dietary recall questionnaires in the NHANES database. The first 24-h dietary recall questionnaires were completed in a mobile examination center (MEC) (Day 1), and the second 24-h dietary recall within 10 days of the MEC assessment was completed by telephone follow-up (Day 2). We used the two days of 24-h dietary recall data to assess the average dietary intake over 2 days. Adherence to the MED was calculated by two steps. First, 24-h dietary recall data was linked to United States Department of Agriculture, Food Patterns Equivalents Database to convert different foods, beverages into equal amounts of food pattern components [31]. In the second step, the alternative MED (aMED) index was used to assess the adherence to the MED [32]. The aMED index is based on the measurement of the intake of different food components, including: total fruits, vegetables (except potatoes), whole grains, legumes, nuts, fish, red and processed meat, ratio of monounsaturated to saturated fat (MUFA/SFA) and alcohol. For a given food component, participants were assigned a score of 1 if the intake was above the median, except for red meat/processed meat and alcohol. One point was assigned for below median intake of red/processed meat and one point for moderate alcohol intake (defined as 10–25 g/day for men and 5–15 g/day for women). A higher total aMED score indicates higher adherence to the MED [33]. The study population was then divided into 3 groups: below median (aMED score 0 ~ 2), median (aMED score = 3), and above median (aMED > 3). Sleep disorders were assessed by a question in sleep questionnaire: “Has your doctor ever told you that you have a sleep disorder?“ (Yes/No)[34]. Sleep duration was assessed through the question: “How much sleep do you get?” (hours, range 0 ~ 12 h/day). Sleep questions including presence of sleep disorders and sleep duration, were obtained from “sleep disorders” questionnaire data set. The questionnaire has been applied and validated in many studies [34, 35]. Trained interviewers utilized the Computer-Assisted Personal Interview (CAPI) system to pose the inquiries, which featured built-in consistency checks to minimize any data entry errors. Additionally, revisions were implemented to guarantee the data’s completeness, consistency, and analytical usefulness. The questionnaire records were scrutinized through audio recordings to confirm their validity. To further clarify whether there is a joint effect of lower adherence to the MED and sleep disorders on mortality, the population was divided into six groups according to aMED levels and sleep disorders: high aMED without sleep disorder, medium aMED without sleep disorder, low aMED without sleep disorder, high aMED with sleep disorder, medium aMED with sleep disorder, and low aMED with sleep disorder.

#### Assessment of other covariates

Baseline and sociodemographic data, laboratory tests, medical condition data, and other dietary recall data were collected from the population. Baseline and sociodemographic data included age, sex, race, education level, marital status, annual household income, family poverty income ratio (PIR), smoking status, body mass index (BMI), and mean systolic/diastolic blood pressure (SBP/DBP). PIR was a ratio of monthly family income to the poverty threshold specific to family size, released by Health and Human Services poverty guideline. Smoking was defined as smoking fewer than 100 cigarettes in life or smoking at the time of survey. Laboratory tests included triglycerides (TG), total cholesterol (TC), low density lipoprotein-cholesterol (LDL-C), high density lipoprotein-cholesterol (HDL-C), fasting glucose, glycated hemoglobin A1c (HbA1c), Homeostatic Model Assessment for Insulin Resistance (HOMA-IR), and estimated glomerular filtration rate (eGFR). History of previous hypertension, diabetes mellitus, congestive heart failure (CHF), coronary heart disease (CHD), and stroke were included from medical condition questionnaires. Previous hypertension was defined as self-reported physician diagnosis of hypertension, or oral antihypertensive drugs or elevated mean blood pressure (systolic ≥ 130 and/or diastolic ≥ 85 mmHg). Diabetes was defined as meeting any of the following items: self-reported physician diagnosis of diabetes, self-reported use of insulin or oral hypoglycemic drugs, a fasting glucose concentration > 126 mg/dL, HbA1c ≥ 6.5%, and Oral Glucose Tolerance Test (OGTT) ≥ 200 mg/dL. The rest of the medical history was directly collected through questionnaires. Other dietary intake variables included total calorie intake, carbohydrate intake, protein intake, fat intake, dietary fiber intake, added sugar intake, monounsaturated fatty acid, polyunsaturated fatty acid, saturated fatty acid intake, electrolytes intake, caffeine intake, and fresh water intake.

#### Assessment of mortality

Enrolled patients were followed up until December 31, 2015. Mortality status, causes of death, and follow-up time were determined based on the NDI, which can be downloaded from NCHS website. Causes of death were coded according to ICD-10.The study outcomes included mortality from all causes, cancer (codes C00-C97), and CVD (codes I00-I09, I11, I13, and I20-I51). The median follow-up time for all patients was 111.6 months.

### Statistical analysis

All analyses were performed with the statistical package R (http://www.R-project.org, R Foundation). The statistical analyses followed the criteria from NHANES, which takes into account the complex sampling design (https://wwwn.cdc.gov/nchs/nhanes/tutorials/sampledesign.aspx). All analyses needed to be weighted to represent the U.S. population. We calculated the weighted estimates according to NHANES analytic guidelines (https://wwwn.cdc.gov/nchs/nhanes/analyticguidelines.aspx#sample-design). Baseline information was described among three aMED groups. Continuous variables are expressed as survey-weighted means (95% CI). Categorical variables were expressed as survey-weighted percentage (95% CI). Differences in continuous variables among groups were analyzed by survey-weighted linear regression (svyglm). Differences in categorical variables were analyzed by survey-weighted Chi-square test (svytable). Cox proportional risk regression models were used to calculate HRs and 95% CIs for sleep disorder, and aMED on all-cause, cardiovascular mortality and cancer mortality. Model I was adjusted for age, sex, and race (Mexican American, non-Hispanic white, non-Hispanic black, other races); model II was further adjusted for education level (below high school, high school or equivalent, college or above), marital status (married, widowed, divorced, and never married), and family PIR; model III was further adjusted for BMI, smoking status, total energy intake, and comorbidities including diabetes mellitus, hypertension, CHF, CHD, and stroke. Patients were further grouped according to aMED and sleep disorder (presence or absence), and Cox proportional risk regression was performed to analyze the relationship with mortality. Interaction effect of sleep disorder with aMED on mortality outcomes was inspected by likelihood ratio test, and two-sided P for interaction of 0.1 were considered statistically significant. Survival curves for the aMED and sleep disorder subgroups were plotted using the Kaplan Meier method and examined by the log-rank test. In addition, restricted cubic spline (RCS) plots were used to assess the dose-response relationship between aMED score and mortality risk. Multiple imputations with chained equations were applied to impute missing values. All analyses were repeated with the complete data to examine the robustness of the results. Two-sided P values of 0.05 were considered statistically significant.

## Results

### Baseline characteristics

A total of 23,212 participants were included in the study. The weighted demographic baseline characteristics of the participants are shown in Table 1. The study population was divided into three groups: high aMED (score 4 to 9), medium aMED (score 3) and low aMED (score 0 to 2). The low aMED group was younger, more Mexican, less educated, had a higher proportion of divorce, higher BMI levels, higher resting SBP levels, a higher proportion of smoking, and a higher proportion of prior strokes or diabetes. The low aMED group had a higher proportion of sleep disorders at 10.5% and shorter nighttime sleep duration (mean 6.8 h). The low aMED group had higher fasting TG levels and lower HDL-C levels. The enrolled population was also divided into 2 groups according to the presence or absence of sleep disorders, and the proportion of sleep disorders in the total population was 8.3% (Table S1). Those with sleep disorders were older, more non-Hispanic, had higher BMI, higher resting blood pressure levels, more comorbidities, shorter sleep duration, higher TG, fasting glucose, HbA1c, HOMA-IR, and lower HDL and eGFR. Table S2 and Table S1 showed intake of individual MED components and other nutrients among different aMED groups or sleep disorder groups. Intake of fruits, vegetables, whole grains, legumes, nuts, seafood, MUFA/SFA and alcohol consumption were lower in the low aMED group. In the sleep disorder group, whole grains, legumes, and alcohol intake were lower, calorie intake from fat was higher, and caffeine intake was higher.

**Table 1:** Baseline demographics among patients with low, medium and high aMED

	Total	aMED ( Above Median)	aMED (Median)	aMED (Below Median)	P
		Score 4–9	Score 3	Score 0–2	
Number of subjects	23,212	13,208	5032	4972	
Age (year)	46.6 (46.0 ,47.2)	47.7 (46.9 ,48.4)	45.6 (44.8 ,46.4)	44.8 (44.0 ,45.6)	< 0.0001
Female	52.5 (51.7 ,53.2)	52.3 (51.2 ,53.4)	51.7 (49.9 ,53.5)	53.9 (51.9 ,55.8)	0.2433
Race					< 0.0001
Mexican American	9.9 (8.4 ,11.7)	7.4 (6.0 ,9.2)	6.1 (4.7 ,7.9)	9.9 (8.4 ,11.7)	
Non-Hispanic White	67.6 (64.5 ,70.5)	69.0 (65.3 ,72.4)	70.8 (66.9 ,74.5)	67.6 (64.5 ,70.5)	
Non-Hispanic Black	9.6 (8.3 ,11.2)	13.7 (11.6 ,16.0)	14.6 (12.4 ,17.1)	9.6 (8.3 ,11.2)	
Other Hispanic	5.0 (4.1 ,6.1)	5.2 (4.0 ,6.6)	4.3 (3.1 ,5.8)	5.0 (4.1 ,6.1)	
Other Race	7.9 (6.8 ,9.1)	4.8 (4.0 ,5.8)	4.2 (3.3 ,5.3)	7.9 (6.8 ,9.1)	
Family PIR	3.0 (2.9 ,3.1)	3.2 (3.1 ,3.3)	2.9 (2.7 ,3.0)	2.5 (2.4 ,2.6)	
Educational level					< 0.0001
Below high school	16.7 (15.3 ,18.1)	14.3 (12.9 ,15.8)	18.1 (16.3 ,19.9)	21.9 (19.5 ,24.5)	
High school Graduate	22.8 (21.6 ,24.0)	19.0 (17.8 ,20.4)	26.5 (24.3 ,28.8)	29.2 (27.1 ,31.3)	
College Graduate or above	60.5 (58.4 ,62.6)	66.7 (64.5 ,68.8)	55.4 (52.6 ,58.2)	48.9 (46.1 ,51.7)	
Marital status					< 0.0001
Married	55.7 (54.0 ,57.4)	60.1 (58.3 ,61.9)	52.9 (50.2 ,55.6)	46.5 (43.8 ,49.3)	
Divorced/Separated/Widowed/Never married	36.9 (35.3 ,38.5)	33.8 (32.1 ,35.4)	38.2 (35.7 ,40.9)	44.0 (41.3 ,46.7)	
Living with partner	7.4 (6.8 ,8.0)	6.1 (5.5 ,6.8)	8.8 (7.6 ,10.2)	9.5 (8.3 ,10.8)	
BMI	28.8 (28.6 ,29.0)	28.3 (28.1 ,28.6)	29.3 (29.0 ,29.7)	29.5 (29.2 ,29.8)	< 0.0001
SBP	121.4 (121.0 ,121.9)	121.1 (120.6 ,121.7)	121.6 (121.0 ,122.3)	122.2 (121.5 ,122.9)	0.0175
DBP	70.4 (69.9 ,70.8)	70.3 (69.9 ,70.8)	70.5 (69.9 ,71.0)	70.4 (69.8 ,71.0)	0.8573
Smoking	45.7 (43.9 ,47.5)	35.7 (33.5 ,38.1)	51.6 (48.6 ,54.7)	60.7 (57.8 ,63.5)	< 0.0001
CHF	2.4 (2.1 ,2.7)	2.2 (1.8 ,2.7)	2.6 (2.1 ,3.2)	2.6 (2.2 ,3.1)	0.2323
CHD	3.3 (2.9 ,3.7)	3.3 (2.8 ,3.9)	3.1 (2.5 ,3.8)	3.5 (2.9 ,4.1)	0.7118
Stroke	2.8 (2.5 ,3.2)	2.3 (2.0 ,2.7)	2.9 (2.3 ,3.6)	4.2 (3.5 ,4.9)	< 0.0001
Diabetes mellitus	12.4 (11.7 ,13.2)	11.7 (10.9 ,12.6)	13.8 (12.3 ,15.4)	12.9 (12.0 ,14.0)	0.0146
Hypertension	40.1 (38.7 ,41.4)	39.4 (37.8 ,41.1)	40.0 (37.8 ,42.3)	41.9 (39.9 ,44.0)	0.1172
Sleep disorder	8.5 (7.9 ,9.0)	7.7 (7.0 ,8.3)	8.7 (7.6 ,10.0)	10.5 (9.1 ,11.9)	0.0003
Sleep hours at night	6.9 (6.9 ,6.9)	7.0 (7.0 ,7.0)	6.9 (6.8 ,6.9)	6.8 (6.7 ,6.9)	< 0.0001
TG (mmol/L)	1.5 (1.4 ,1.5)	1.4 (1.4 ,1.5)	1.5 (1.5 ,1.6)	1.5 (1.5 ,1.6)	0.0077
TC (mmol/L)	5.1 (5.0 ,5.1)	5.1 (5.0 ,5.1)	5.1 (5.0 ,5.1)	5.1 (5.0 ,5.1)	0.697
HDL-C (mmol/L)	1.4 (1.4 ,1.4)	1.4 (1.4 ,1.4)	1.3 (1.3 ,1.4)	1.3 (1.3 ,1.3)	< 0.0001
LDL-C (mmol/L)	3.0 (2.9 ,3.0)	3.0 (2.9 ,3.0)	3.0 (2.9 ,3.1)	3.0 (2.9 ,3.0)	0.3511
Fasting Glucose (mmol/L)	5.8 (5.8 ,5.9)	5.8 (5.7 ,5.9)	5.9 (5.8 ,6.0)	5.8 (5.7 ,5.9)	0.1823
HbA1c, %	5.6 (5.5 ,5.6)	5.6 (5.5 ,5.6)	5.6 (5.6 ,5.6)	5.6 (5.5 ,5.6)	0.1507
HOMA-IR	3.6 (3.5 ,3.8)	3.5 (3.2 ,3.7)	3.7 (3.4 ,3.9)	4.0 (3.7 ,4.2)	0.0601
eGFR, mL/min	98.8 (98.0 ,99.6)	99.0 (98.1 ,100.0)	98.6 (97.1 ,100.0)	98.1 (97.0 ,99.3)	0.3393

aMED, alternative Mediterranean diet; PIR, poverty income ratio; BMI, body mass index; SBP, systolic pressure; DBP, diastolic pressure; CHF, congestive heart failure; CHD, coronary heart disease; TG, triglycerides; TC, total cholesterol; HDL-C, high density lipoprotein-cholesterol; LDL-C, low density lipoprotein-cholesterol; HbA1c, glycated hemoglobin A1c; HOMA-IR, Homeostatic Model Assessment for Insulin Resistance; eGFR, estimated glomerular filtration rate. Data are presented as mean (95% CI) or percentage (95% CI). For continuous variables, p value was by survey-weighted linear regression (svyglm). For categorical variables, p value was by survey-weighted Chi-square test (svytable)

### Association between aMED, sleep disorder and mortality

During a median follow-up of 111.6 months, there were 3082 deaths, of which 755 (24.5%) were of cardiovascular causes and 722 (23.4%) were due to cancer. Cox proportional risk regression models were used to calculate the association of aMED, sleep disorders with all-cause mortality, and cause-specific mortality. After fully adjusting for covariates (model III), it suggested that lower aMED significantly increased the risk of all-cause mortality (HR, 1.42, 95% CI, 1.20–1.67, P < 0.0001) and cardiovascular mortality (HR, 1.13, 95% CI, 1.05–1.51, P = 0.0422), compared with higher aMED group. When further analyzing aMED as a continuous variable, a lower aMED score was associated with a higher risk of all-cause mortality (HR, 1.09 aMED per 1 decrease, 95% CI, 1.04–1.15, P = 0.0005). The presence of sleep disorders significantly increased the risk of all-cause death and cardiovascular mortality (HR, 1.42, 95% CI, 1.15–1.75, P = 0.001; HR, 1.61, 95% CI, 1.10–2.35, P = 0.0148). However, lower aMED did not significantly increase the risk of cancer mortality (HR, 1.21, 95% CI, 0.78–1.87, P = 0.3947), and sleep disorders did not significantly increase the risk of cancer mortality either (HR, 1.04, 95% CI, 0.64–1.68, P = 0.8804) (Table 2).

**Table 2:** Association between aMED, sleep disorder and mortality

	Unadjusted		Model I		Model II		Model III	
	HR (95% CI)	P value	HR (95% CI)	P value	HR (95% CI)	P value	HR (95% CI)	P value
**All-cause mortality**								
Higher aMED	Reference							
Median aMED	1.25 (1.10–1.44)	0.001	1.49 (1.30–1.71)	< 0.0001	1.35 (1.16–1.57)	0.0001	1.36 (1.14–1.63)	0.0006
Lower aMED	1.50 (1.32–1.69)	< 0.0001	1.77 (1.58–1.98)	< 0.0001	1.45 (1.27–1.66)	< 0.0001	1.42 (1.20–1.67)	< 0.0001
aMED(continuous)	1.12 (1.08–1.16)	< 0.0001	1.18 (1.15–1.22)	< 0.0001	1.11 (1.07–1.15)	< 0.0001	1.09 (1.04–1.15)	0.0005
Sleep disorder*	1.63 (1.39–1.91)	< 0.0001	1.41 (1.21–1.63)	< 0.0001	1.49 (1.26–1.75)	< 0.0001	1.42 (1.15–1.75)	0.0011
**CVD mortality**								
Higher aMED	Reference							
Median aMED	1.19 (0.95–1.49)	0.1275	1.44 (1.14–1.82)	0.0025	1.27 (0.98–1.66)	0.0703	1.05 (0.94–1.50)	0.1023
Lower aMED	1.35 (1.07–1.70)	0.0107	1.59 (1.27-2.00)	< 0.0001	1.28 (1.05–1.65)	0.0321	1.13 (1.05–1.51)	0.0422
aMED(continuous)	1.10 (1.03–1.18)	0.0035	1.17 (1.09–1.25)	< 0.0001	1.09 (1.01–1.17)	0.0341	0.99 (0.85–1.15)	0.8960
Sleep disorder*	1.84 (1.34–2.51)	0.0001	1.66 (1.22–2.27)	0.0013	1.83 (1.36–2.47)	< 0.0001	1.61 (1.10–2.35)	0.0148
**Cancer mortality**								
Higher aMED	Reference							
Median aMED	0.99 (0.68–1.43)	0.9377	1.16 (0.81–1.66)	0.427	1.07 (0.73–1.56)	0.7411	1.32 (0.79–2.21)	0.29
Lower aMED	1.16 (0.91–1.47)	0.2457	1.35 (1.07–1.72)	0.0132	1.20 (0.93–1.55)	0.1713	1.21 (0.78–1.87)	0.3947
aMED(continuous)	1.04 (0.99–1.11)	0.1424	1.10 (1.04–1.17)	0.0007	1.06 (0.99–1.13)	0.0920	1.07 (0.96–1.19)	0.2454
Sleep disorder*	1.11 (0.79–1.55)	0.5493	0.93 (0.67–1.30)	0.6736	0.98 (0.69–1.39)	0.9166	1.04 (0.64–1.68)	0.8804

CVD, cardiovascular disease; Model I initially was adjusted for age, sex, and race (Mexican American, non-Hispanic white, non-Hispanic black, other races); model II was further adjusted for education level (below high school, high school or equivalent, college or above), marital status (married, widowed, divorced, and never married), and family PIR based on model I; model III further adjusted for BMI, smoking status, total energy intake, comorbidities including diabetes mellitus, hypertension, CHF, CHD, and stroke. *No sleep disorder was set as the reference group

### Interaction of the MED and sleep disorders

**Fig. 2:**
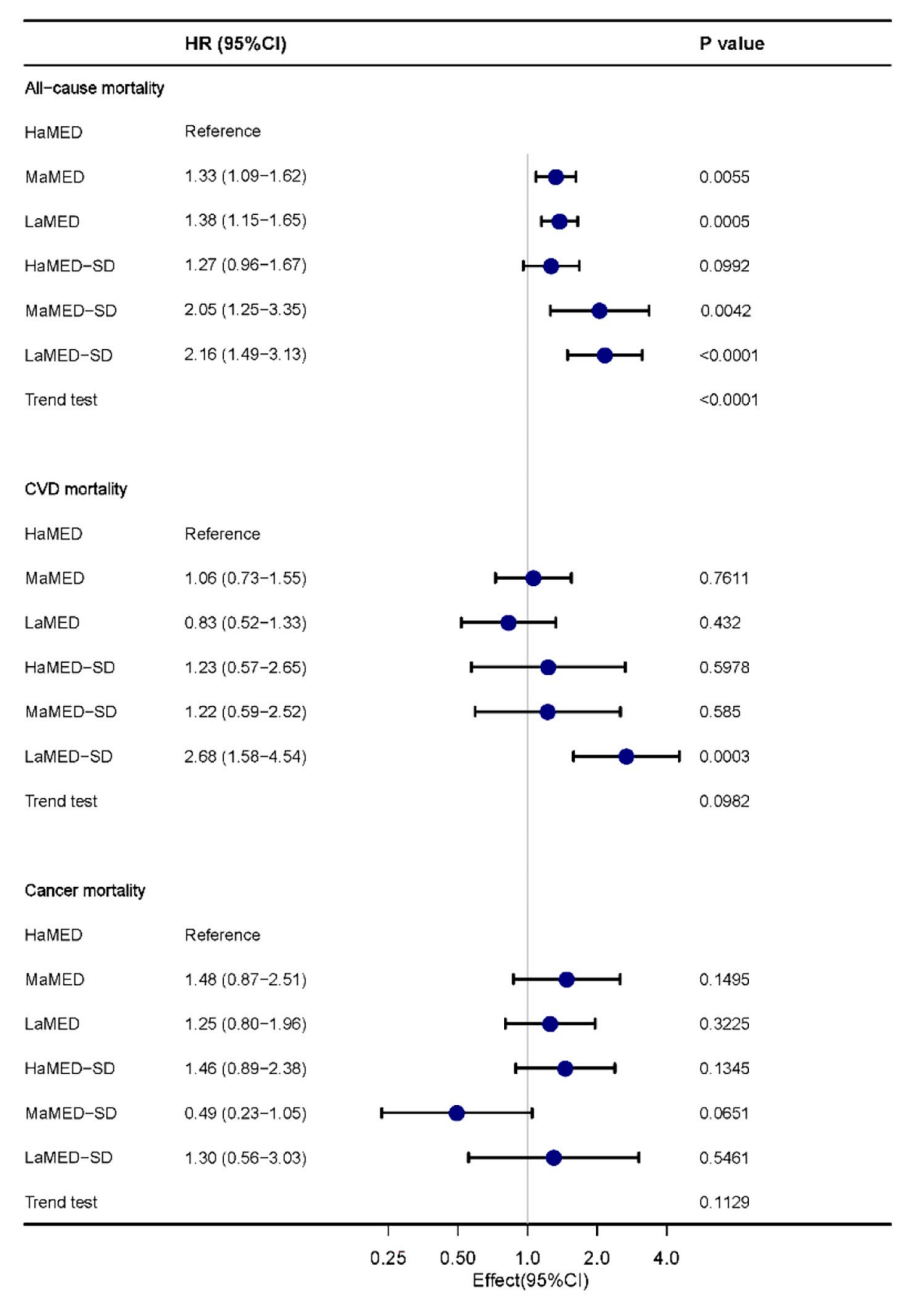
Forest plot of association between aMED*sleep disorder and mortality. HaMED, high aMED (score 4 ~ 9) without sleep disorder; MaMED, medium aMED (score 3) without sleep disorder; LaMED, low aMED (score 0 ~ 2) without sleep disorder; HaMED-SD, high aMED with sleep disorder; MaMED-SD, medium aMED with sleep disorder; LaMED-SD, low aMED with sleep disorder. Model was adjusted for age, sex, race (Mexican American, non-Hispanic white, non-Hispanic black, other races), education level (below high school, high school or equivalent, college or above), marital status (married, widowed, divorced, and never married), family PIR, BMI, smoking status, total energy intake, comorbidities including diabetes mellitus, hypertension, CHF, CHD, and stroke

**Fig. 3:**
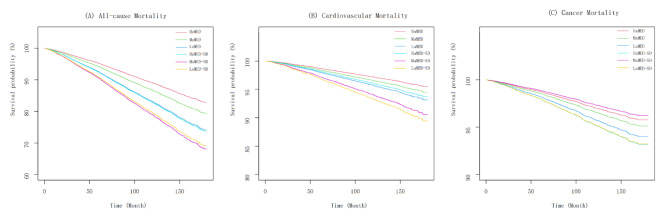
Survival curves for different aMED*sleep disorder groups. HaMED, high aMED (score 4 ~ 9) without sleep disorder; MaMED, medium aMED (score 3) without sleep disorder; LaMED, low aMED (score 0 ~ 2) without sleep disorder; HaMED-SD, high aMED with sleep disorder; MaMED-SD, medium aMED with sleep disorder; LaMED-SD, low aMED with sleep disorder

The joint effect between lower aMED and sleep disorder on long-term mortality was found. People with lower aMED combined with sleep disorder had the highest risk of all-cause and cardiovascular mortality (HR, 2.16, 95% CI 1.49–3.13, p for trend < 0.0001; HR, 2.68, 95% CI 1.58–4.54, p for trend = 0.0982) (Fig. 2). The interaction between aMED grouping and sleep disorder on mortality was further investigated. There was a significant interaction effect between aMED and sleep disorders on cardiovascular mortality (p for interaction = 0.033), while no significant interaction was found between aMED and sleep disorders for all-cause mortality and cancer mortality (p for interaction = 0.184 and 0.955 respectively) (Table 3). In addition, Fig. 3 suggested a higher cumulative risk of all-cause mortality and cumulative cardiovascular mortality for those with low to medium levels of aMED combined with sleep disorders during follow-up (both p < 0.0001). Similar trend was not found in the cancer mortality analysis.

**Table 3:** Interaction analysis of aMED and sleep disorder on mortality

aMED categories	Sleep Disorder	HR 95% CI	P value	P for interaction
**All-cause mortality**				0.184
Higher aMED	Without	1(Ref)		
Median aMED	Without	1.27 (1.11 ~ 1.46)	< 0.001	
Lower aMED	Without	1.38 (1.21 ~ 1.58)	< 0.001	
Higher aMED	With	1(Ref)		
Median aMED	With	1.28 (0.9 ~ 1.82)	0.173	
Lower aMED	With	1.46 (1.02 ~ 2.1)	0.04	
**CVD mortality**				0.033
Higher aMED	Without	1(Ref)		
Median aMED	Without	1.19 (0.9 ~ 1.58)	0.216	
Lower aMED	Without	1.08 (0.81 ~ 1.43)	0.607	
Higher aMED	With	1(Ref)		
Median aMED	With	1.26 (0.56 ~ 2.86)	0.576	
Lower aMED	With	1.65 (0.75 ~ 3.61)	0.213	
**Cancer mortality**				0.955
Higher aMED	Without	1(Ref)		
Median aMED	Without	1.28 (0.98 ~ 1.67)	0.066	
Lower aMED	Without	1.46 (1.13 ~ 1.88)	0.004	
Higher aMED	With	1(Ref)		
Median aMED	With	0.68 (0.3 ~ 1.54)	0.35	
Lower aMED	With	1.48 (0.72 ~ 3.04)	0.285	

Adjusted for age, sex, race (Mexican American, non-Hispanic white, non-Hispanic black, other races), education level (below high school, high school or equivalent, college or above), marital status (married, widowed, divorced, and never married), family PIR, BMI, smoking status, total energy intake, comorbidities including diabetes mellitus, hypertension, CHF, CHD, and stroke

### Sensitivity analysis

**Fig. 4:**
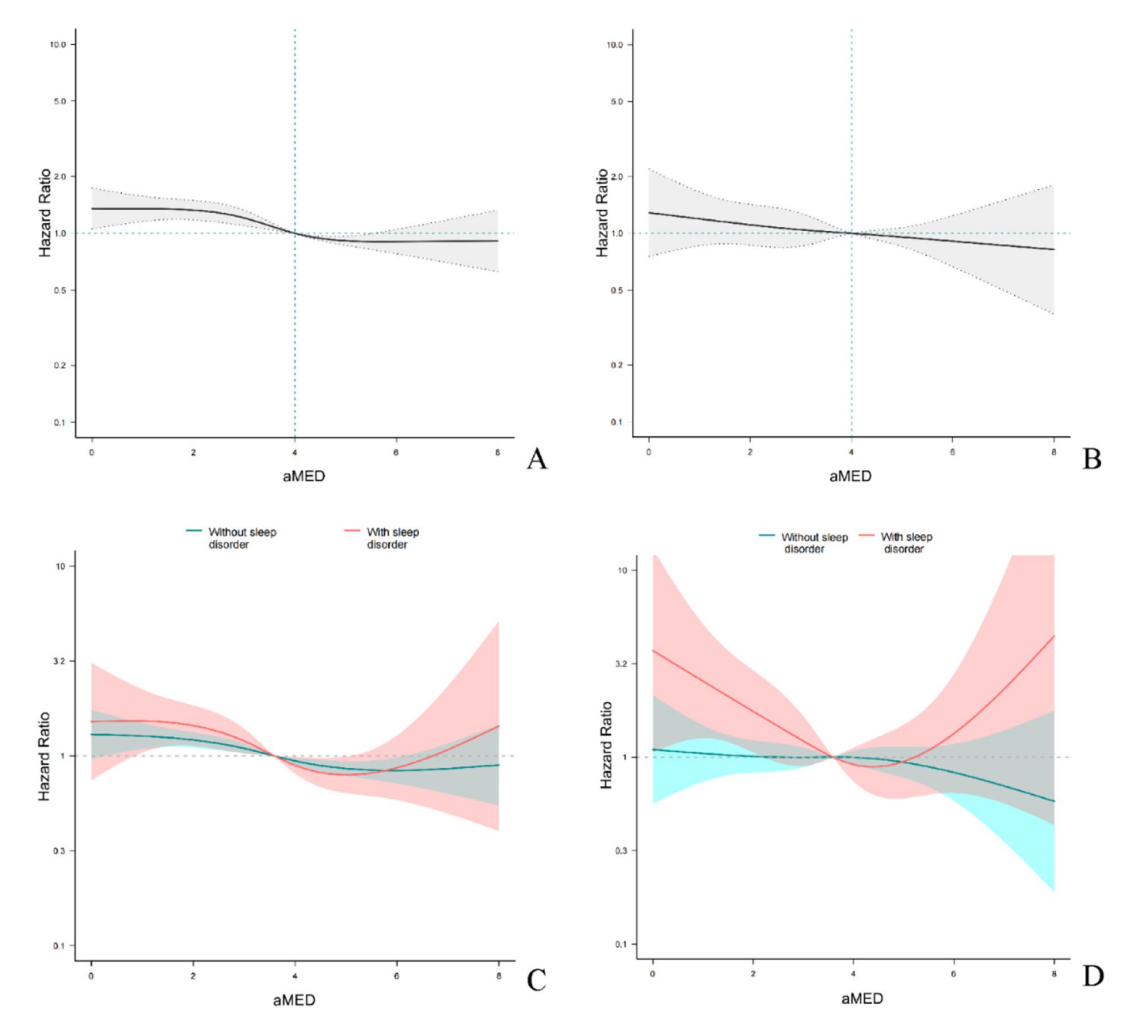
Restricted cubic spline plots of the dose-response relationship between aMED and mortality **A.** Dose-response relationship between aMED and all-cause mortality; **B.** Dose-response relationship between aMED and CVD mortality; **C.** Dose-response relationship between aMED and all-cause mortality in participants with or without sleep disorder; **D.** Dose-response relationship between aMED and CVD mortality in participants with or without sleep disorder. The model was adjusted for age, sex, race (Mexican American, non-Hispanic white, non-Hispanic black, other races), education level (below high school, high school or equivalent, college or above), marital status (married, widowed, divorced, and never married), family PIR, BMI, smoking status, total energy intake, comorbidities including diabetes mellitus, hypertension, CHF, CHD, and stroke

The dose-response relationship between aMED and all-cause mortality and cardiovascular mortality was further assessed by RCS curves (Fig. 4), and a monotonic dose-response relationship between aMED and all-cause mortality was found. A similar linear relationship was found between aMED and cardiovascular mortality. When aMED was less than 4, the tendency for the increased risk of cardiovascular mortality was more pronounced in the population with sleep disorders. In addition, we assessed whether there was an interaction between different food components of the MED and sleep disorders (Table S3). A significant interaction between intake of whole grains and sleep disorders for all-cause mortality (p for interaction = 0.001) was found, and there were also interactions for fruits, whole grains, and legumes with sleep disorders for cardiovascular mortality (p for interaction = 0.088, 0.078, and 0.077 respectively). In addition, alcohol consumption was found to interact with sleep disorders for cardiovascular mortality (p for interaction = 0.022), and moderate alcohol consumption was not associated with a reduced risk of cardiovascular death in the sleep disordered population. Subgroup analyses were performed to reveal specific populations more susceptible to MED or sleep disorders (Table S4). Lower aMED was not associated with all-cause mortality in those with combined CHF, CHD, stroke, or eGFR < 60 mL/min. Among those aged < 65 years, women, BMI < 30, nonsmokers, and those with comorbid hypertension or CHD, sleep disorders were not associated with all-cause mortality. In order to more accurately assess the joint effect between aMED (a dietary score) and sleep disorders on long-term mortality outcome, we excluded patients who died early (during the first two year of follow-up). The results showed that even after excluding early death events, the aforementioned interaction remained stable (Table S5 and S6).

## Discussion

This study confirms for the first time that the combined presence of poor adherence to the MED and sleep disorders synergistically increases all-cause and cardiovascular mortality, and there was a significant interaction effect between aMED and sleep disorders on cardiovascular mortality, with a higher relative risk of mortality among those with poor adherence to the MED and sleep disorders.

A large number of studies have examined the correlation between diet habits, sleep disorders, and mortality. Poor dietary habits are strongly associated with the risk of developing chronic diseases and long-term mortality [36]. Poor dietary patterns with high intake of sodium, carbohydrate, intake of excessive processed foods, and low intake of fruits and vegetables are key risk factors for long-term mortality, and cardiovascular events, cancer incidence may mediate the causal relationship between diet and mortality [37]. A MED style with abundant intakes of vegetables and fruits, fish, grains, legumes, and olive oil has been shown to reduce the risk of cardiovascular events, cancer death, and all-cause mortality [38]. Studies have found that the protective effect of the MED against mortality may be related to improvement of lipid and glucose metabolism and reduced levels of systemic inflammation [39]. A study published in Lancet in May 2022 found that people on the MED had a lower risk of major cardiovascular events compared to those on a low-fat diet alone [40]. This additional benefit may be related to the specific antioxidant components of the MED such as vitamin E, resveratrol, and curcumin, as well as anti-inflammatory components such as polyphenols, polyphenols, and dietary fiber [41, 42]. Sleep disorders such as insomnia and lack of sleep duration are also strongly associated with the incidence of cardiovascular events. Sleep disorders can cause excessive activation of the sympathetic nervous system, leading to elevated cortisol levels, elevated glucose and blood pressure, reduced heart rate variability, and elevated levels of inflammatory markers [43]. Thus, there was a broad impact of sleep disorders on disease development and prognosis, such as cardiovascular and cerebrovascular events, cognitive decline, and cancer occurrence [16, 20, 44]. With NHANES data representing different ethnic groups across the United States, our study preliminarily verified that both sleep disorders and poor adherence to the MED significantly increased the risk of all-cause mortality and cardiovascular mortality. However, we did not find a significant association between these two factors and the risk of cancer mortality. The reason for this may be that some cancer patients have comorbid cardiovascular diseases and there is a competing risk relationship between cardiovascular mortality and cancer mortality, leading to statistical bias. In addition, it has been reported in the literature that individuals lack of sleep have a 3-fold increased risk of dying from cancer among those with a history of heart disease or stroke, while this trend was not found in healthy populations [45]. Thus, the risk of cancer-related death may be more significant in those with specific cardiovascular diseases.

Meanwhile, MED and sleep disorders are closely related and influence each other. Our analysis found a significant interaction between aMED and sleep disorders for cardiovascular mortality. For people with sleep disorder, the risk of cardiovascular death was significantly higher with decreased aMED scores, while this trend was not found in the population without sleep disorders. The MED may have a more prominent role in contributing to cardiovascular events in the sleep-disordered population. A previous cohort study also confirmed that adequate sleep, in addition to maintaining a traditional healthy lifestyle such as a healthy diet and regular physical activity, can additionally reduce the risk of cardiovascular disease [46]. This may be because the systemic inflammatory response is more active in the context of sleep disorders. Studies have found that sleep disorders increase the levels of systemic inflammatory markers such as leukocytes, cytokines, interleukins, C-reactive protein, etc.[47]. This leads to a significant beneficial effect of the MED featuring anti-inflammatory properties, reducing the risk of atherosclerosis, development of cardiovascular disease, and thus associated mortality [48]. Our study also found that this beneficial effect has limitation. After an aMED score greater than 4, the effect of the MED in reducing the risk of cardiovascular mortality became limited in the sleep-disordered population. This may be due to the fact that sleep disorders increase chronic metabolic disturbances, sympathetic hyperactivation, triggering hypertension, and other cardiovascular diseases, and the presence of these comorbidities counteracts the benefit of the MED. Our subgroup analysis also found that adherence to the MED was not effective in reducing mortality risk in people with CHF, CHD, and stroke, indicating that the effect of MED on improving the prognosis of cardiovascular diseases hardly exceeded the significant protective effect of pharmacological or interventional treatments. Therefore, overemphasizing the benefits of the MED is also one-sided.

Our study further explored which dietary components play a key role in the relationship between the MED, sleep disorders, and mortality outcomes. Fruits, whole grains, legumes, and alcohol consumption significantly interacted with sleep disorder for cardiovascular mortality. Studies confirmed that people with sleep disorders are often deficient in tryptophan, melatonin, B vitamins, calcium, and trace elements such as selenium and magnesium [49, 50]. Whole grains are rich in magnesium and selenium as well as B vitamins [51]. Diets high in magnesium have been shown to be associated with a reduction in daytime sleepiness in women [52]. Those with low dietary selenium levels are more likely to experience depression and insomnia [53] The association between vitamin B deficiency and sleep disorders has also been widely demonstrated [54]. Legumes are rich in tryptophan and calcium, and calcium deficiency is associated with reduced deep sleep, while tryptophan, a raw material for the synthesis of endogenous melatonin, can improve sleep quality. All of these foods help to improve sleep disorders and may also reduce the risk of cardiovascular events [55, 56]. Adequate supplementation with magnesium, selenium, and B vitamins are important for the prevention of atherosclerotic disease [57, 58]. We also found that moderate alcohol consumption increased the risk of cardiovascular death in people with sleep disorders. This is consistent with the findings of previous studies. First, any level of alcohol consumption reduces the quality of sleep [59] and also increases the risk of cardiovascular disease [60]. The benefits of small amounts of alcohol consumption on cardiovascular disease observed in previous studies are due to the fact that the effects of different lifestyles on outcomes were not excluded. Based on a UK biobank study confirming that even small amounts of alcohol consumption increase the risk of cardiovascular disease [61]. Therefore, adherence to a MED with fruits, whole grains, and legumes has positive implications for people with sleep disorders.

The strengths of the study can be summarized as follows: This is the first study investigating the joint effect between adherence to MED and sleep disorders on long-term mortality. To strengthen the reliability of the findings, potential covariates were adjusted, and the aMED was treated as continuous and categorical variables. By applying a quantified score to assess the adherence of MED, we explored the non-linear relationship between aMED and mortality outcomes, and found that in the population with sleep disorders, the risk of death significantly increases when aMED is less than 4.

Our study has some limitations. Limited by the study design of NHANES, the assessment of sleep disorders was only from self-reported questionnaires, without investigating objective sleep patterns, and medical records were lacking, which may have introduced risk of misclassification of sleep disorders, and hindered further analysis of sleep disorder-related parameters, such as insomnia severity, excessive daytime sleepiness, and sleep apnea. In addition, despite efforts to minimize data collection bias, there may have been some discrepancies between the reported dietary information and participants’ actual habits, which could have potentially influenced our results. Meanwhile, information about medical treatment for sleep disorder was lacking. which was not adjusted for in our analyses.

## Conclusions

Poor adherence to the MED and sleep disorders jointly increased long-term all-cause mortality and cardiovascular mortality in the U.S. population. There was a significant interaction effect between aMED and sleep disorders on cardiovascular mortality.

## Electronic supplementary material

Below is the link to the electronic supplementary material.

Supplementary Material 1

## Data Availability

The datasets generated and/or analyzed during the current study are available in the NHANES website (https://www.cdc.gov/nchs/nhanes/index.htm).

## Abbreviations

MED: Mediterranean diet
NHANES: National Health and Nutrition Examination Survey
aMED: alternative Mediterranean diet
CVD: cardiovascular disease
NCHS: National Center for Health Statistics
MUFA/SFA: monounsaturated to saturated fat
BMI: body mass index
SBP: systolic blood pressure
DBP: diastolic blood pressure
TG: triglycerides
TC: total cholesterol
LDL-C: low density lipoprotein-cholesterol
HDL-C: high density lipoprotein-cholesterol
HbA1c: glycated hemoglobin A1c
HOMA-IR: Homeostatic Model Assessment for Insulin Resistance
CHF: congestive heart failure
CHD: coronary heart disease
