# Supplementary material for: Sleep disorder, Mediterranean diet, and all-cause and cause-specific mortality: a prospective cohort study
Source: BMC Public Health. 2023 May 18;23:904. doi: 10.1186/s12889-023-15870-x (PMC10193722; doi:10.1186/s12889-023-15870-x)
Supplement: Supplementary file 1 — Supplementary Material 1 [file 12889_2023_15870_MOESM1_ESM.docx]

**Supplementary Tables**

**Table S1 Baseline demographics between patients with or without sleep mortality.**

|  | Total | With sleep disorder | Without sleep disorder | P |
| --- | --- | --- | --- | --- |
| Number of subjects | 23212 | 1926 | 21286 |  |
| Age (year) | 46.6 (46.0 ,47.2) | 51.6 (50.7 ,52.4) | 46.2 (45.5 ,46.8) | <0.0001 |
| Female | 52.5 (51.7 ,53.2) | 49.3 (45.9 ,52.8) | 52.8 (52.0 ,53.6) | 0.0533 |
| Race |  |  |  | <0.0001 |
| Mexican American | 8.6 (7.2 ,10.2) | 4.8 (3.5 ,6.5) | 8.9 (7.5 ,10.5) | |
| Non-Hispanic White | 68.6 (65.4 ,71.5) | 74.1 (70.2 ,77.6) | 68.0 (64.9 ,71.0) | |
| Non-Hispanic Black | 11.6 (10.0 ,13.4) | 11.7 (9.6 ,14.2) | 11.5 (10.0 ,13.4) | |
| Other Hispanic | 4.9 (3.9 ,6.0) | 4.8 (3.6 ,6.5) | 4.9 (3.9 ,6.0) | |
| Other Race | 6.4 (5.7 ,7.3) | 4.6 (3.5 ,6.0) | 6.6 (5.8 ,7.5) | |
| Family PIR | 3.0 (2.9 ,3.1) | 2.9 (2.7 ,3.1) | 3.0 (2.9 ,3.1) | 0.1572 |
| Educational level |  |  |  | 0.1397 |
| Below high school | 16.7 (15.3 ,18.1) | 14.8 (12.3 ,17.7) | 16.9 (15.5 ,18.3) | |
| High school Graduate | 22.8 (21.6 ,24.0) | 24.7 (21.7 ,28.1) | 22.6 (21.4 ,23.8) | |
| College Graduate or above | 60.5 (58.4 ,62.6) | 60.4 (56.7 ,64.1) | 60.5 (58.4 ,62.6) | |
| Marital status |  |  |  | 0.1585 |
| Married | 55.7 (54.0 ,57.4) | 58.0 (54.0 ,61.9) | 55.5 (53.8 ,57.2) | |
| Divorced/Separated/Widowed/Never married | 36.9 (35.3 ,38.5) | 36.1 (32.4 ,40.0) | 36.9 (35.3 ,38.6) | |
| Living with partner | 7.4 (6.8 ,8.0) | 5.9 (4.6 ,7.5) | 7.6 (6.9 ,8.2) | |
| BMI | 28.8 (28.6 ,29.0) | 32.6 (32.0 ,33.2) | 28.4 (28.3 ,28.6) | <0.0001 |
| SBP | 121.4 (121.0 ,121.9) | 123.5 (122.3 ,124.7) | 121.3 (120.8 ,121.7) | 0.0007 |
| DBP | 70.4 (69.9 ,70.8) | 71.1 (70.3 ,71.9) | 70.3 (69.9 ,70.7) | 0.0474 |
| Smoking | 45.7 (43.9 ,47.5) | 44.4 (39.6 ,49.4) | 45.8 (44.0 ,47.6) | 0.5805 |
| Congestive heart failure | 2.4 (2.1 ,2.7) | 6.1 (5.0 ,7.4) | 2.0 (1.8 ,2.3) | <0.0001 |
| Coronary heart disease | 3.3 (2.9 ,3.7) | 8.7 (7.1 ,10.6) | 2.8 (2.5 ,3.2) | <0.0001 |
| Stroke | 2.8 (2.5 ,3.2) | 6.1 (4.8 ,7.7) | 2.5 (2.2 ,2.9) | <0.0001 |
| Diabetes mellitus | 12.4 (11.7 ,13.2) | 23.5 (20.6 ,26.7) | 11.4 (10.7 ,12.1) | <0.0001 |
| Hypertension | 40.1 (38.7 ,41.4) | 57.6 (54.1 ,61.1) | 38.5 (37.0 ,39.9) | <0.0001 |
| Sleep hours at night | 6.9 (6.9 ,6.9) | 6.5 (6.4 ,6.6) | 7.0 (6.9 ,7.0) | <0.0001 |
| Triglyceride (mmol/L) | 1.5 (1.4 ,1.5) | 1.7 (1.5 ,1.8) | 1.5 (1.4 ,1.5) | 0.002 |
| Total cholesterol (mmol/L) | 5.1 (5.0 ,5.1) | 5.0 (4.9 ,5.1) | 5.1 (5.0 ,5.1) | 0.0828 |
| HDL-Cholesterol (mmol/L) | 1.4 (1.4 ,1.4) | 1.3 (1.2 ,1.3) | 1.4 (1.4 ,1.4) | <0.0001 |
| LDL-cholesterol (mmol/L) | 3.0 (2.9 ,3.0) | 2.9 (2.8 ,3.0) | 3.0 (2.9 ,3.0) | 0.1296 |
| Fasting Glucose (mmol/L) | 5.8 (5.8 ,5.9) | 6.3 (6.1 ,6.5) | 5.8 (5.7 ,5.8) | <0.0001 |
| HbA1c, % | 5.6 (5.5 ,5.6) | 5.8 (5.8 ,5.9) | 5.5 (5.5 ,5.6) | <0.0001 |
| HOMA-IR | 3.6 (3.5 ,3.8) | 5.2 (4.7 ,5.7) | 3.5 (3.3 ,3.6) | <0.0001 |
| eGFR, mL/min | 98.8 (98.0 ,99.6) | 93.0 (91.4 ,94.6) | 99.3 (98.5 ,100.1) | <0.0001 |
| aMED | 3.8 (3.8 ,3.9) | 3.6 (3.5 ,3.7) | 3.8 (3.8 ,3.9) | 0.0005 |
| aMED categories |  |  |  | 0.0003 |
| aMED ( Above Median) | 57.2 (55.8 ,58.5) | 51.6 (48.2 ,55.0) | 57.7 (56.2 ,59.1) | |
| aMED (Median) | 22.0 (21.2 ,22.8) | 22.7 (20.0 ,25.6) | 21.9 (21.1 ,22.8) | |
| aMED (Below Median) | 20.8 (19.7 ,22.0) | 25.7 (22.7 ,28.9) | 20.4 (19.2 ,21.6) | |
| Fruits (cup) | 1.0 (1.0 ,1.0) | 0.9 (0.9 ,1.0) | 1.0 (1.0 ,1.1) | 0.0711 |
| Vegetables (cup) | 1.1 (1.1 ,1.2) | 1.1 (1.0 ,1.2) | 1.1 (1.1 ,1.2) | 0.0681 |
| Whole grains (gm) | 185.5 (183.3 ,187.7) | 179.8 (174.1 ,185.4) | 186.0 (183.7 ,188.4) | 0.0476 |
| Legumes (gm) | 16.3 (15.3 ,17.3) | 13.9 (12.0 ,15.9) | 16.5 (15.5 ,17.5) | 0.0114 |
| Nuts (gm) | 19.6 (18.6 ,20.7) | 20.8 (18.1 ,23.4) | 19.5 (18.4 ,20.6) | 0.3517 |
| Seafood (gm) | 18.4 (17.0 ,19.8) | 16.9 (14.2 ,19.6) | 18.6 (17.1 ,20.0) | 0.2383 |
| Meat (gm) | 76.7 (74.9 ,78.5) | 79.2 (74.1 ,84.3) | 76.5 (74.7 ,78.4) | 0.3163 |
| Alcohol (gm) | 9.0 (8.5 ,9.6) | 6.6 (5.4 ,7.8) | 9.3 (8.7 ,9.8) | 0.0001 |
| MUFA/SFA | 1.1 (1.1 ,1.2) | 1.1 (1.1 ,1.2) | 1.1 (1.1 ,1.2) | 0.2933 |
| Total calorie, kcal | 2100.2 (2080.1 ,2120.2) | 2061.2 (1997.3 ,2125.1) | 2103.8 (2083.3 ,2124.3) | 0.2033 |
| % from carbohydrate | 49.5 (49.3 ,49.7) | 48.7 (47.9 ,49.4) | 49.6 (49.3 ,49.8) | 0.0245 |
| % from protein | 16.3 (16.2 ,16.5) | 16.3 (16.0 ,16.6) | 16.3 (16.2 ,16.5) | 0.9622 |
| % from fat | 34.2 (34.0 ,34.4) | 35.0 (34.3 ,35.6) | 34.1 (33.9 ,34.3) | 0.0101 |
| Fiber (gm) | 16.9 (16.7 ,17.2) | 16.5 (15.9 ,17.1) | 17.0 (16.7 ,17.3) | 0.0818 |
| PUFA | 17.9 (17.6 ,18.1) | 17.9 (17.2 ,18.5) | 17.9 (17.6 ,18.1) | 0.9841 |
| Cholesterol (mg) | 284.9 (281.0 ,288.8) | 280.4 (268.5 ,292.4) | 285.3 (281.3 ,289.3) | 0.4342 |
| Calcium (mg) | 965.9 (952.2 ,979.5) | 953.2 (917.5 ,988.9) | 967.1 (953.2 ,980.9) | 0.4436 |
| Phosphorus (mg) | 1371.9 (1357.4 ,1386.5) | 1346.3 (1300.7 ,1391.9) | 1374.3 (1360.1 ,1388.6) | 0.2165 |
| Magnesium (mg) | 300.7 (296.7 ,304.8) | 295.0 (285.2 ,304.8) | 301.2 (297.3 ,305.1) | 0.1604 |
| Sodium (mg) | 3484.5 (3452.1 ,3516.9) | 3450.0 (3341.5 ,3558.6) | 3487.7 (3454.4 ,3521.1) | 0.5107 |
| Potassium (mg) | 2702.9 (2672.2 ,2733.6) | 2670.8 (2589.6 ,2752.1) | 2705.8 (2676.2 ,2735.4) | 0.355 |
| Caffeine (mg) | 164.6 (158.2 ,171.1) | 178.6 (165.0 ,192.2) | 163.3 (156.7 ,169.9) | 0.0283 |
| Plain water (gm) | 1038.7 (1005.1 ,1072.2) | 1041.7 (980.2 ,1103.2) | 1038.4 (1004.4 ,1072.4) | 0.9095 |

aMED, alternative Mediterranean diet; PIR, poverty income ratio; BMI, body mass index; SBP, systolic pressure; DBP, diastolic pressure; CHF, congestive heart failure; CHD, coronary heart disease; TG, triglycerides; TC, total cholesterol; HDL-C, high density lipoprotein-cholesterol; LDL-C, low density lipoprotein-cholesterol; HbA1c, glycated hemoglobin A1c; HOMA-IR, Homeostatic Model Assessment for Insulin Resistance; eGFR, estimated glomerular filtration rate. Data are presented as mean (95% CI) or percentage (95% CI). For continuous variables, p value was by survey-weighted linear regression (svyglm). For categorical variables, p value was by survey-weighted Chi-square test (svytable).

**Table S2** **Component of Mediterranean diet and other nutrients per day in different aMED groups**

|  | Total | aMED ( Above Median) | aMED (Median) | aMED (Below Median) | P |
| --- | --- | --- | --- | --- | --- |
|  |  | Score 4–9 | Score 3 | Score 0-2 |  |
| Fruits (cup) | 1.0 (1.0 ,1.0) | 1.3 (1.3 ,1.3) | 0.8 (0.8 ,0.9) | 0.4 (0.4 ,0.5) | <0.0001 |
| Vegetables (cup) | 1.1 (1.1 ,1.2) | 1.4 (1.4 ,1.4) | 1.0 (0.9 ,1.0) | 0.6 (0.6 ,0.6) | <0.0001 |
| Whole grains (gm) | 185.5 (183.3 ,187.7) | 204.3 (201.7 ,206.9) | 177.8 (173.5 ,182.2) | 142.1 (138.3 ,145.9) | <0.0001 |
| Legumes (gm) | 16.3 (15.3 ,17.3) | 22.9 (21.5 ,24.4) | 10.3 (9.0 ,11.5) | 4.4 (3.7 ,5.2) | <0.0001 |
| Nuts (gm) | 19.6 (18.6 ,20.7) | 28.5 (26.9 ,30.1) | 11.4 (10.2 ,12.6) | 3.9 (3.2 ,4.6) | <0.0001 |
| Seafood (gm) | 18.4 (17.0 ,19.8) | 26.6 (24.6 ,28.6) | 11.0 (9.1 ,12.9) | 3.8 (2.9 ,4.7) | <0.0001 |
| Meat (gm) | 76.7 (74.9 ,78.5) | 66.4 (64.2 ,68.5) | 89.2 (86.2 ,92.2) | 92.0 (88.8 ,95.1) | <0.0001 |
| Alcohol (gm) | 9.0 (8.5 ,9.6) | 9.1 (8.5 ,9.7) | 9.2 (8.2 ,10.2) | 8.7 (7.8 ,9.6) | 0.7223 |
| MUFA/SFA | 1.1 (1.1 ,1.2) | 1.2 (1.2 ,1.2) | 1.1 (1.1 ,1.1) | 1.0 (1.0 ,1.0) | <0.0001 |
| Total calorie, kcal | 2100.2 (2080.1 ,2120.2) | 2224.1 (2201.7 ,2246.6) | 2067.2 (2034.0 ,2100.3) | 1795.1 (1764.7 ,1825.6) | <0.0001 |
| % from carbohydrate | 49.5 (49.3 ,49.7) | 49.9 (49.7 ,50.1) | 48.9 (48.5 ,49.4) | 48.9 (48.4 ,49.4) | <0.0001 |
| % from protein | 16.3 (16.2 ,16.5) | 16.2 (16.1 ,16.3) | 16.4 (16.2 ,16.6) | 16.7 (16.4 ,17.0) | 0.0048 |
| % from fat | 34.2 (34.0 ,34.4) | 33.9 (33.6 ,34.1) | 34.7 (34.3 ,35.0) | 34.4 (34.1 ,34.8) | 0.0012 |
| Fiber (gm) | 16.9 (16.7 ,17.2) | 20.3 (20.0 ,20.6) | 14.5 (14.2 ,14.8) | 10.4 (10.1 ,10.7) | <0.0001 |
| PUFA | 17.9 (17.6 ,18.1) | 20.0 (19.7 ,20.3) | 16.7 (16.3 ,17.1) | 13.2 (12.9 ,13.5) | <0.0001 |
| Cholesterol (mg) | 284.9 (281.0 ,288.8) | 290.2 (285.4 ,295.0) | 290.2 (282.7 ,297.8) | 264.6 (256.1 ,273.1) | <0.0001 |
| Calcium (mg) | 965.9 (952.2 ,979.5) | 1022.4 (1008.2 ,1036.6) | 942.8 (921.6 ,964.1) | 835.3 (809.0 ,861.6) | <0.0001 |
| Phosphorus (mg) | 1371.9 (1357.4 ,1386.5) | 1469.2 (1454.6 ,1483.8) | 1327.8 (1306.0 ,1349.6) | 1151.9 (1125.0 ,1178.9) | <0.0001 |
| Magnesium (mg) | 300.7 (296.7 ,304.8) | 341.5 (337.5 ,345.5) | 271.7 (267.2 ,276.1) | 219.4 (214.5 ,224.4) | <0.0001 |
| Sodium (mg) | 3484.5 (3452.1 ,3516.9) | 3700.3 (3663.5 ,3737.1) | 3428.6 (3361.8 ,3495.4) | 2951.8 (2892.5 ,3011.2) | <0.0001 |
| Potassium (mg) | 2702.9 (2672.2 ,2733.6) | 2982.9 (2953.0 ,3012.8) | 2535.9 (2489.5 ,2582.3) | 2111.3 (2060.9 ,2161.8) | <0.0001 |
| Caffeine (mg) | 164.6 (158.2 ,171.1) | 160.0 (152.8 ,167.1) | 167.2 (157.3 ,177.1) | 174.6 (163.8 ,185.4) | 0.0346 |
| Plain water (gm) | 1038.7 (1005.1 ,1072.2) | 1130.2 (1091.8 ,1168.5) | 984.9 (935.3 ,1034.4) | 844.6 (802.1 ,887.0) | <0.0001 |

aMED, alternative Mediterranean diet; MUFA/SFA, ratio of monounsaturated to saturated fat; PUFA, polyunsaturated fatty acid. Data are presented as mean (95% CI) or percentage (95% CI). For continuous variables, p value was by survey-weighted linear regression (svyglm). For categorical variables, p value was by survey-weighted Chi-square test (svytable).

**Table S3 Interaction effects of aMED components and sleep disorder on mortality.**

| aMED individual score | Sleep Disorder | adj.HR, 95% CI | adjusted P value | P for interaction |
| --- | --- | --- | --- | --- |
| **All-cause mortality** |  |  |  |  |
| Fruits score = 0 (reference=1) | Without | 1.06 (0.95~1.18) | 0.332 | 0.41 |
|  | With | 1.06 (0.78~1.43) | 0.719 |  |
| Vegetables score = 0 (reference=1) | Without | 1.24 (1.11~1.39) | <0.001 | 0.34 |
|  | With | 0.95 (0.71~1.28) | 0.744 |  |
| Whole grains score = 0 (reference=1) | Without | 1 (0.88~1.13) | 0.94 | 0.001 |
|  | With | 1.57 (1.11~2.22) | 0.011 |  |
| Legumes score = 0 (reference=1) | Without | 1.2 (1.06~1.35) | 0.005 | 0.474 |
|  | With | 1.32 (0.95~1.83) | 0.093 |  |
| Nuts score = 0 (reference=1) | Without | 1.27 (1.14~1.42) | <0.001 | 0.172 |
|  | With | 1.41 (1.04~1.91) | 0.025 |  |
| Sea food score = 0 (reference=1) | Without | 1.16 (1.03~1.3) | 0.014 | 0.387 |
|  | With | 1.04 (0.76~1.44) | 0.801 |  |
| Meat score = 0 (reference=1) | Without | 0.96 (0.86~1.07) | 0.439 | 0.237 |
|  | With | 0.75 (0.56~1.03) | 0.073 |  |
| Alcohol score = 0 (reference=1) | Without | 0.7 (0.37~1.33) | 0.278 | 0.15 |
|  | With | 0.13 (0.03~0.66) | 0.014 |  |
| MUFA/SFA score = 0 (reference=1) | Without | 1.12 (1.01~1.25) | 0.035 | 0.516 |
|  | With | 1.06 (0.8~1.41) | 0.69 |  |
| **CVD mortality** |  |  |  |  |
| Fruits score = 0 (reference=1) | Without | 1.06 (0.84~1.34) | 0.634 | 0.088 |
|  | With | 1.27 (0.65~2.46) | 0.487 |  |
| Vegetables score = 0 (reference=1) | Without | 0.99 (0.78~1.25) | 0.917 | 0.197 |
|  | With | 1.21 (0.62~2.36) | 0.583 |  |
| Whole grains score = 0 (reference=1) | Without | 0.81 (0.62~1.05) | 0.113 | 0.078 |
|  | With | 1.14 (0.53~2.48) | 0.733 |  |
| Legumes score = 0 (reference=1) | Without | 1.16 (0.9~1.51) | 0.256 | 0.077 |
|  | With | 2.51 (1.1~5.72) | 0.028 |  |
| Nuts score = 0 (reference=1) | Without | 1.22 (0.96~1.54) | 0.1 | 0.158 |
|  | With | 1.46 (0.74~2.86) | 0.275 |  |
| Sea food score = 0 (reference=1) | Without | 1.18 (0.92~1.5) | 0.198 | 0.134 |
|  | With | 0.73 (0.38~1.42) | 0.352 |  |
| Meat score = 0 (reference=1) | Without | 1.06 (0.84~1.34) | 0.642 | 0.36 |
|  | With | 0.69 (0.35~1.34) | 0.268 |  |
| Alcohol score = 0 (reference=1) | Without | Inf (0~Inf) | 0.988 | 0.022 |
|  | With | 0.03 (0~0.43) | 0.01 |  |
| MUFA/SFA score = 0 (reference=1) | Without | 1.03 (0.82~1.29) | 0.819 | 0.222 |
|  | With | 0.8 (0.42~1.53) | 0.498 |  |
| **Cancer mortality** |  |  |  |  |
| Fruits score = 0 (reference=1) | Without | 1.4 (1.12~1.74) | 0.003 | 0.654 |
|  | With | 1.86 (0.99~3.48) | 0.052 |  |
| Vegetables score = 0 (reference=1) | Without | 1.17 (0.94~1.45) | 0.158 | 0.089 |
|  | With | 0.6 (0.32~1.14) | 0.118 |  |
| Whole grains score = 0 (reference=1) | Without | 1.06 (0.83~1.35) | 0.655 | 0.228 |
|  | With | 1.61 (0.79~3.28) | 0.187 |  |
| Legumes score = 0 (reference=1) | Without | 1.1 (0.87~1.39) | 0.441 | 0.575 |
|  | With | 1.36 (0.69~2.7) | 0.373 |  |
| Nuts score = 0 (reference=1) | Without | 1.17 (0.94~1.45) | 0.154 | 0.62 |
|  | With | 1.47 (0.78~2.76) | 0.234 |  |
| Sea food score = 0 (reference=1) | Without | 1.12 (0.89~1.41) | 0.322 | 0.403 |
|  | With | 0.84 (0.44~1.59) | 0.592 |  |
| Meat score = 0 (reference=1) | Without | 1.07 (0.86~1.34) | 0.535 | 0.312 |
|  | With | 0.88 (0.65~1.23) | 0.210 |  |
| Alcohol score = 0 (reference=1) | Without | 0.57 (0.21~1.58) | 0.282 | 0.591 |
|  | With | Inf (0~Inf) | 0.999 |  |
| MUFA/SFA score = 0 (reference=1) | Without | 1.11 (0.9~1.37) | 0.323 | 0.659 |
|  | With | 1.29 (0.71~2.32) | 0.402 |  |

Adjusted for age, sex, race (Mexican American, non-Hispanic white, non-Hispanic black, other races), education level (below high school, high school or equivalent, college or above), marital status (married, widowed, divorced, and never married), family PIR, BMI, smoking status, total energy intake, comorbidities including diabetes mellitus, hypertension, CHF, CHD, and stroke.

**Table S4 Subgroup analysis.**

|  | aMED and all-cause mortality | | | |  | Sleep disorder and all-cause mortality | | |
| --- | --- | --- | --- | --- | --- | --- | --- | --- |
| Subgroup | | HR 95%CI | P value | P for interaction |  | HR 95%CI | P value | P for interaction |
| Age<65 years | | 1.06 (0.99~1.12) | 0.08 | 0.674 |  | 1.25 (0.98~1.6) | 0.068 | 0.283 |
| Age≥65 years | | 1.12 (1.07~1.17) | <0.001 |  |  | 1.32 (1.08~1.61) | 0.006 |  |
| Male | | 1.09 (1.04~1.13) | <0.001 | 0.304 |  | 1.35 (1.13~1.63) | 0.001 | 0.543 |
| Female | | 1.13 (1.06~1.2) | <0.001 |  |  | 1.15 (0.87~1.51) | 0.331 |  |
| BMI<30 | | 1.09 (1.05~1.14) | <0.001 | 0.414 |  | 1.23 (0.97~1.55) | 0.089 | 0.336 |
| BMI≥30 | | 1.11 (1.04~1.18) | 0.001 |  |  | 1.25 (1.02~1.53) | 0.034 |  |
| Smoking | | 1.07 (1.01~1.14) | 0.025 | 0.362 |  | 1.36 (1.13~1.63) | 0.001 | 0.833 |
| No smoking | | 1.11 (1.06~1.16) | <0.001 |  |  | 1.19 (0.9~1.56) | 0.223 |  |
| Hypertension | | 1.1 (1.06~1.15) | <0.001 | 0.526 |  | 1.35 (0.98~1.87) | 0.063 | 0.479 |
| No hypertension | | 1.11 (1.03~1.19) | 0.005 |  |  | 1.23 (1.03~1.46) | 0.021 |  |
| Diabetes | | 1.1 (1.03~1.17) | 0.003 | 0.511 |  | 1.24 (1.01~1.52) | 0.044 | 0.593 |
| No diabetes | | 1.1 (1.03~1.17) | <0.001 |  |  | 1.34 (1.07~1.68) | 0.012 |  |
| CHF | | 1.06 (0.95~1.18) | 0.28 | 0.040 |  | 1.49 (1.05~2.12) | 0.026 | 0.181 |
| No CHF | | 1.11 (1.07~1.15) | <0.001 |  |  | 1.22 (1.03~1.45) | 0.023 |  |
| CHD | | 1.07 (0.98~1.17) | 0.157 | 0.218 |  | 1.37 (0.98~1.92) | 0.068 | 0.713 |
| No CHD | | 1.1 (1.06~1.15) | <0.001 |  |  | 1.25 (1.05~1.48) | 0.011 |  |
| Stroke | | 1.07 (0.96~1.18) | 0.212 | 0.588 |  | 1.64 (1.11~2.41) | 0.013 | 0.695 |
| No stroke | | 1.1 (1.06~1.14) | <0.001 |  |  | 1.23 (1.04~1.45) | 0.015 |  |
| eGFR<60 mL/min | | 1.05 (0.97~1.13) | 0.234 | 0.046 |  | 1.59 (1.13~2.23) | 0.007 | 0.260 |
| eGFR≥60 mL/min | | 1.11 (1.06~1.15) | <0.001 |  |  | 1.24 (1.03~1.49) | 0.021 |  |

CHF, congestive heart failure; CHD, coronary heart disease; eGFR, estimated glomerular filtration rate;

Adjusted for age, sex, race (Mexican American, non-Hispanic white, non-Hispanic black, other races), education level (below high school, high school or equivalent, college or above), marital status (married, widowed, divorced, and never married), family PIR, BMI, smoking status, total energy intake, comorbidities including diabetes mellitus, hypertension, CHF, CHD, and stroke.

**Table S5 Association between aMED*sleep disorder and mortality excluding the death population during the first 2 years of follow-up.**

| **Variable** | **n.event (%)** | **crude HR (95% CI)** | **crude P value** | **adj.HR (95% CI)** | **adj.P value** |
| --- | --- | --- | --- | --- | --- |
| **All-cause mortality** | |  |  |  |  |
| HaMED | 1191 (9.9) | 1(Ref) |  | 1(Ref) |  |
| MaMED | 518 (11.6) | 1.17 (1.05~1.29) | 0.004 | 1.23 (1.06~1.41) | 0.005 |
| LaMED | 636 (14.6) | 1.51 (1.37~1.66) | <0.001 | 1.29 (1.12~1.48) | <0.001 |
| HaMED-SD | 139 (14.4) | 1.54 (1.29~1.84) | <0.001 | 1.19 (0.94~1.51) | 0.156 |
| MaMED-SD | 77 (17.8) | 1.97 (1.56~2.48) | <0.001 | 1.66 (1.24~2.22) | 0.001 |
| LaMED-SD | 68 (14.7) | 1.69 (1.32~2.15) | <0.001 | 1.74 (1.27~2.38) | 0.001 |
| Trend test |  |  |  |  | <0.001 |
|  |  |  |  |  |  |
| **CVD mortality** |  |  |  |  |  |
| aMEDSLQ1 | 294 (2.4) | 1(Ref) |  | 1(Ref) |  |
| aMEDSLQ2 | 132 (2.9) | 1.2 (0.98~1.48) | 0.077 | 1.17 (0.88~1.57) | 0.279 |
| aMEDSLQ3 | 144 (3.3) | 1.39 (1.13~1.69) | 0.001 | 0.95 (0.7~1.29) | 0.744 |
| aMEDSLQ4 | 29 (3) | 1.3 (0.89~1.91) | 0.176 | 0.89 (0.52~1.52) | 0.659 |
| aMEDSLQ5 | 22 (5.1) | 2.27 (1.47~3.49) | <0.001 | 1.62 (0.9~2.9) | 0.105 |
| aMEDSLQ6 | 21 (4.5) | 2.1 (1.35~3.27) | 0.001 | 1.76 (0.96~3.22) | 0.068 |
| Trend test |  |  |  |  | 0.047 |
|  |  |  |  |  |  |
| **Cancer mortality** |  |  |  |  |  |
| aMEDSLQ1 | 289 (2.4) | 1(Ref) |  | 1(Ref) |  |
| aMEDSLQ2 | 115 (2.6) | 1.07 (0.86~1.32) | 0.563 | 1.23 (0.92~1.63) | 0.161 |
| aMEDSLQ3 | 145 (3.3) | 1.42 (1.16~1.73) | 0.001 | 1.38 (1.05~1.82) | 0.02 |
| aMEDSLQ4 | 38 (3.9) | 1.73 (1.24~2.43) | 0.001 | 1.47 (0.94~2.3) | 0.09 |
| aMEDSLQ5 | 7 (1.6) | 0.74 (0.35~1.56) | 0.422 | 0.83 (0.37~1.89) | 0.66 |
| aMEDSLQ6 | 12 (2.6) | 1.22 (0.68~2.17) | 0.5 | 1.46 (0.74~2.89) | 0.28 |
| Trend test |  |  |  |  | 0.058 |

HaMED, high aMED (score 4~9) without sleep disorder; MaMED, medium aMED (score 3) without sleep disorder; LaMED, low aMED (score 0~2) without sleep disorder; HaMED-SD, high aMED with sleep disorder; MaMED-SD, medium aMED with sleep disorder; LaMED-SD, low aMED with sleep disorder. Model was adjusted for age, sex, race (Mexican American, non-Hispanic white, non-Hispanic black, other races), education level (below high school, high school or equivalent, college or above), marital status (married, widowed, divorced, and never married), family PIR, BMI, smoking status, total energy intake, comorbidities including diabetes mellitus, hypertension, CHF, CHD, and stroke.

**Table S6 Interaction analysis of aMED and sleep disorder on mortality excluding the death population during the first 2 years of follow-up.**

| **aMED categories** | **Sleep Disorder** | **HR 95% CI** | **P value** | **P for interaction** |
| --- | --- | --- | --- | --- |
| **All-cause mortality** |  |  |  | 0.525 |
| Higher aMED | Without | 1(Ref) |  |  |
| Median aMED | Without | 1.24 (1.07~1.43) | 0.003 |  |
| Lower aMED | Without | 1.32 (1.15~1.52) | <0.001 |  |
| Higher aMED | With | 1(Ref) |  |  |
| Median aMED | With | 1.32 (0.9~1.92) | 0.152 |  |
| Lower aMED | With | 1.25 (0.83~1.87) | 0.289 |  |
| **CVD mortality** |  |  |  | 0.044 |
| Higher aMED | Without | 1(Ref) |  |  |
| Median aMED | Without | 1.19 (0.89~1.6) | 0.238 |  |
| Lower aMED | Without | 0.99 (0.73~1.34) | 0.946 |  |
| Higher aMED | With | 1(Ref) |  |  |
| Median aMED | With | 1.71 (0.77~3.81) | 0.189 |  |
| Lower aMED | With | 1.4 (0.59~3.32) | 0.441 |  |
| **Cancer mortality** |  |  |  | 0.423 |
| Higher aMED | Without | 1(Ref) |  |  |
| Median aMED | Without | 1.23 (0.93~1.64) | 0.149 |  |
| Lower aMED | Without | 1.41 (1.07~1.85) | 0.015 |  |
| Higher aMED | With | 1(Ref) |  |  |
| Median aMED | With | 0.51 (0.2~1.31) | 0.162 |  |
| Lower aMED | With | 0.9 (0.38~2.13) | 0.817 |  |
